# Comparative efficiency and residue levels of spraying programs against powdery mildew in grape varieties

**DOI:** 10.1515/biol-2025-1144

**Published:** 2025-08-05

**Authors:** Ayşegül Kaya, Himmet Tezcan, Arif Atak

**Affiliations:** Graduate School of Natural and Applied Sciences, Bursa Uludağ University, 16059, Bursa, Turkey; Department of Plant Protection, Agriculture Faculty, Bursa Uludağ University, 16059, Bursa, Turkey; Department of Horticulture, Agriculture Faculty, Bursa Uludağ University, 16059, Bursa, Turkey

**Keywords:** *Vitis vinifera*, powdery mildew control, protection, pesticide residue levels, sustainable viticulture

## Abstract

Powdery mildew (*Erysiphe necator* Schw.) fungal disease in vineyards is becoming an increasingly important concern due to climate change and the emergence of resistant populations after heavy spraying. Recently, new disease control methods based on phenological development, disease development, and meteorological data have been established in Türkiye. These models can provide sufficient protection with minimal fungicide use and minimize residue problems due to excessive fungicide use. In this study, the activities of the UC Davis risk index model, decision support strategy (DSS) models, and classical model, based on plant phenological development stages suggested by the Turkish Ministry of Agriculture and Forestry, were used to protect against powdery mildew. Three alternative control strategies were investigated for 2 years using two grape varieties. In addition, the amount of pesticide residue in the final product by the spraying models was also evaluated. The UC Davis risk index model was applicable in places with similar climatic conditions, such as Bursa Province, and showed better results than other spraying programs. Although the classical model proposed by the Ministry of Agriculture and Forestry is generally less effective than the UC Davis risk index model, once less spraying achieved high effectiveness rates each season. The UC Davis risk index model considerably lowered the disease incidence rate in clusters below the 5% limit. The DSS model provided poorer protection than the other two models evaluated in this study. Among the fungicides with active ingredients, thiophanate-methyl, kresoxim-methyl, and penconazole, only thiophanate-methyl exceeded the 0.1 ppm (mg/kg) limit specified in the European Union Pesticide Maximum Residue Limits and Turkish Food Codex Pesticide Maximum Residue Limits for both grape varieties. Alternating fungicides with different active ingredients instead of a single fungicide at regular intervals throughout the season can reduce residue problems of grapes and the risk of pathogen resistance to fungicides.

## 1 Introduction

Grape (*Vitis vinifera* L.) is a fruit that has been grown in many places in the world for many years and has different forms of evaluation [1]. Viticulture is one of the most important branches of agricultural activity in the world. According to the current data of the Food and Agriculture Organization of the United Nations [2], nearly 75 million tons of grapes are produced worldwide, and Türkiye is among the world’s leading grape producers, accounting for approximately 5.5% of the total production, with 4.16 million tons. In Türkiye, a large part of the production is focused on the cultivation of dry and table grapes, and very intensive spraying is carried out, especially for fungal diseases, during the production season. Particularly, in recent years, due to increasing disease pressure caused by climate change, the desired results cannot be obtained despite the use of intensive pesticide programs, especially for grapes grown for table purposes. In addition, this situation causes serious residual risks for both human and environmental health and the formation of resistant populations [3]. Although there are problems caused by various diseases and pests in grape production, the main factor in yield and quality loss is fungal diseases [4].

The most economically important disease in vineyards worldwide is *Erysiphe necator* Schw. It is a vineyard powdery mildew disease caused by the fungus [5]. *E. necator*, an obligate parasite, can infect all green tissues and bunches of plants. In early fruit infections, the growth of berries stops; in late-stage infections, the berries crack, which significantly reduces the market value of the grapes [6]. Appropriate humidity and temperature conditions accelerate the development of fungal diseases, which cause severe damage to many parts of the vine, including its leaves and fruits. Particularly, the Marmara and Black Sea regions of Türkiye have very suitable climates for these fungal diseases [7].

Problems such as loss of yield and quality in the product as a result of powdery mildew disease in grape cultivation, environmental pollution due to excessive use of fungicides in certain periods based on phenological development stages in control against the disease, and the emergence of pesticide residues in food products (table grapes, vine leaves, raisins, wine, etc.) necessitate the development of more effective methods. In this context, various studies have been conducted around the world, especially based on early warning models, and recommendations for control the disease have been developed. The most important of these models are the degree-day model [8], the UC Davis risk index model (Gubler-Thomas Program) [9], the Oidiag-System 2.2 model [10], and the decision support strategy (DSS) model [11]. In vineyards protected against powdery mildew, spraying methods based on fixed intervals and early warning systems are mainly used, taking into account the phenological development stages. However, pesticides used before harvest and during the storage period can sometimes cause residue problems, and as a result problems are often experienced in terms of the maximum residue limits (MRLs) accepted in domestic and foreign trade. Particularly in foreign trade, exported grape products whose MRL exceeds the acceptable limit are not accepted in recipient countries and are returned [12]. For this reason, the level of residue should also be considered when deciding suitable spray programs for methods to prevent powdery mildew in grapes. Most pesticides are very toxic in nature and pose acute risks to human health and the environment. They cause serious threats to human health, such as diabetes, reproductive disorders, neurological dysfunction, cancer, and respiratory disorders [13]. Also, the wide variety of potential microorganisms that might be used for biological protection and promotion of plant growth includes fungi of the *Trichoderma* genus and species of the genus *Bacillus* [14,15,16,17].

Powdery mildew is a fungal disease that has been increasing its effect in recent years and causes major crop losses in grapes. Various pesticides are used with many different spraying programs to combat this disease. It is reported by researchers that some of these pesticides are not very effective and may leave a residual risk [18,19,20]. In this study, the effectiveness of different spraying programs used in the world regarding powdery mildew in preventing the disease and the possibility of leaving a residual risk were primarily investigated. In line with this objective, our study attempted to determine the most effective spraying program (the UC Davis risk index model, DSS model, and phenology-based standard spraying model – classical model) that could protect two different table grape varieties produced in Bursa Province from powdery mildew disease without posing any risk of chemical residue.

## 2 Materials and methods

### 2.1 Materials

**Figure 1:**
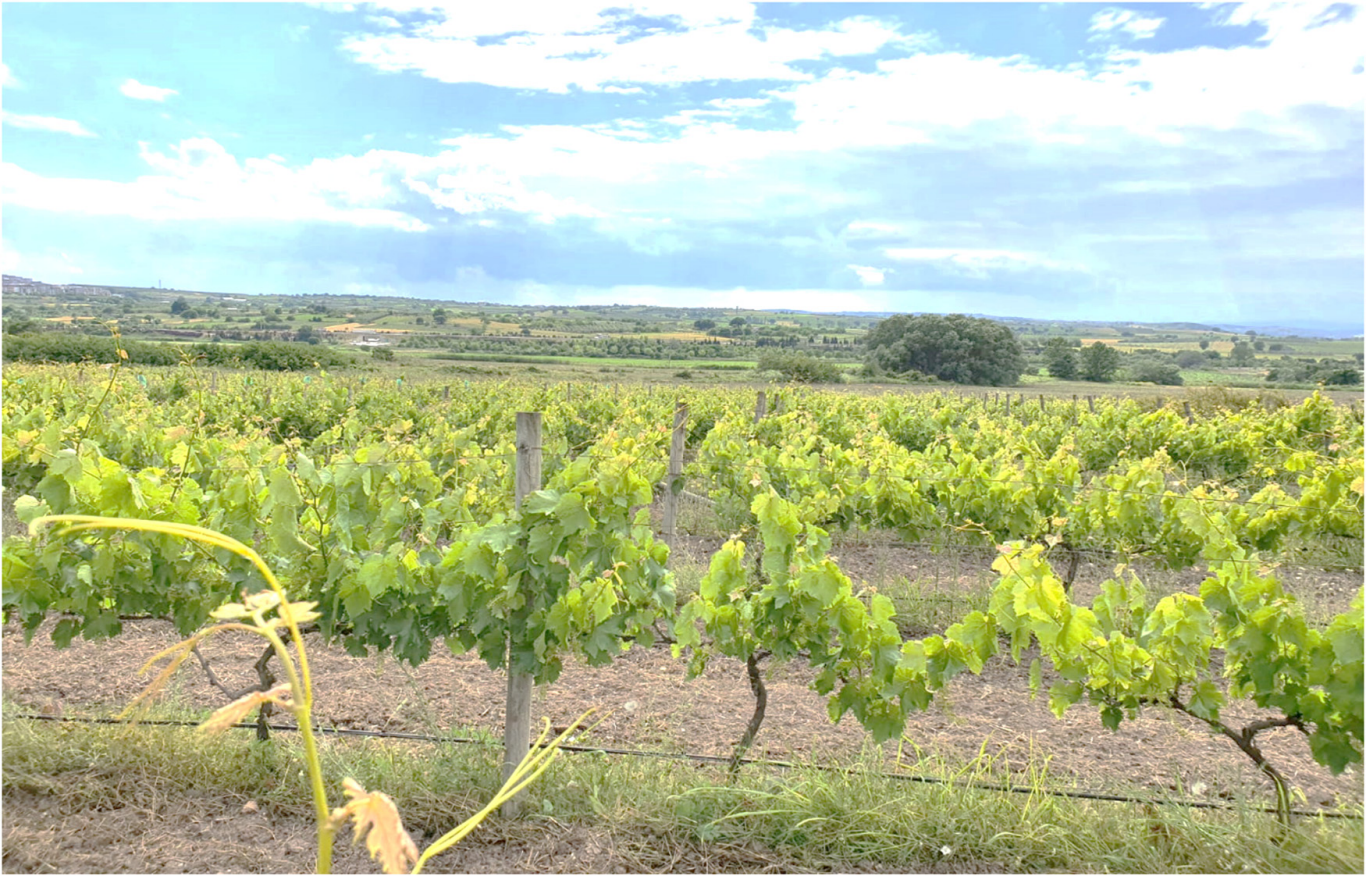
Photo of the vineyard where the experimental area is located.

**Figure 2:**
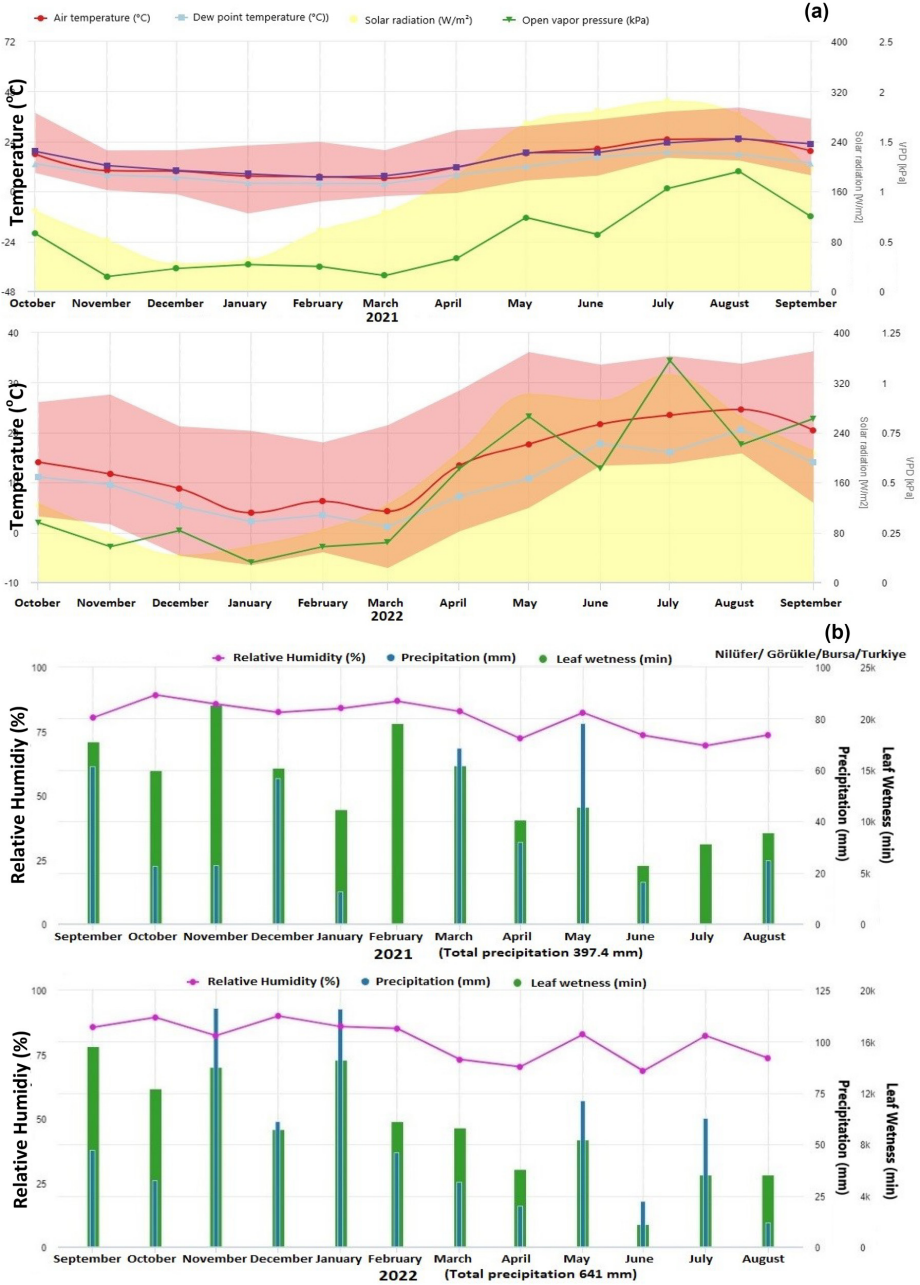
Temperature (^o^C) (a) and relative humidity (%) (b) data received from climate stations in the experimental area in 2021 and 2022.

Using 4-year-old Michele Palieri and Muscat Hamburg varieties grafted on 1103 Paulsen rootstock, the investigation was conducted in a 3 m × 2 m long vineyard situated in the trial region of the Bursa Uludağ University Görükle/Nilüfer campus (Figure 1). The GPS coordinates of the experimental area were 40°14′14.3″N 28°51′35.2″E. The vineyards were formed via a typical wall wire training method. Figure 2a and b shows the important climate data for the experimental area for 2021 and 2022.

Fungicides licensed in Turkiye or other countries for the control of diseases, including Bordeaux slurry, metallic copper, sulfur, kresoxim-methyl, penconazole, and thiophonate-methyl, were used in varieties during the 2021 and 2022 growing seasons as part of the study’s spray program. No fungicide was applied to the control plots. Furthermore, fungicides for vineyard powdery mildew in the Michele Palieri and Muscat Hamburg grape varieties were chosen and applied using the UC Davis model, classical model, and DSS model as guidelines. The commercial names, active ingredients, application doses, and spraying times of the fungicides used in the spraying models are given in Tables 1–3. The doses recommended by the companies producing the fungicides were considered during spraying.

**Table 1:** Fungicides used in the Classic model spraying program in 2021–2022 and the number of applications

Spray model	Number of spray	2021		2022	
		Date of spraying	Name of the fungicide (ingredient and dosage)	Date of spraying	Name of the fungicide (ingredient and dosage)
Classic model	First spraying	April 14	Burgundy slurry	April 25	Burgundy slurry
			(20% metallic copper)		(20% metallic copper)
			500 g/100 L		500 g/100 L
	Second spraying	April 30	Copper	May 24	Topas
					(100 g/L Penconazole)
			500 g/100 L		25 mL/100 L
	Third spraying	May 17	Topas	June 10	Prostar
			(100 g/L Penconazole)		(%50 Kresoxim-ethyl)
			25 mL/100 L		20 mL/100 L
	Fourth spraying	June 3	Prostar	24 June	Topas
			(%50 Kresoxim-ethyl)		(100 g/L Penconazole)
			20 mL/100 L		25 mL/100 L
	Fifth spraying	June 22	Topas	July 8	Prostar
			(100 g/L Penconazole)		(%50 Kresoxim-ethyl)
			25 mL/100 L		20 mL/100 L
	Sixth spraying	July 9	Prostar	July 21	Topas
			(%50 Kresoxim-ethyl)		(100 g/L Penconazole)
			20 mL/100 L		25 mL/100 L
	Seventh spraying	July 22	Rage	August 8	Rage
			(%70 Thiophanate-ethyl)		(%70 Thiophanate-ethyl)
			100 g/100 L		100 g/100 L

**Table 2:** Fungicides used in the UC Davis model spraying program in 2021–2022 and application numbers

Spray model	Number of spray	2021	2022	2021	2022
		Date of spraying	Name of the fungicide (ingredient and dosage)	Date of spraying	Name of the fungicide (ingredient and dosage)
UC Davis model	First spraying	April 14	Burgundy slurry	April 25	Burgundy slurry
			(20% metallic copper)		(20% metallic copper)
			500 g/100 L		500 g/100 L
	Second spraying	April 30	Copper	May 12	Sulfur (73% S)
			500 g/100 L		500 g/100 L
	Third spraying	May 7	Sulfur (73% S)	May 17	Topas
					(100 g/L Penconazole)
			500 g/100 L		25 mL/100 L
	Fourth spraying	May 17	Topas	June 10	Prostar
			(100 g/L Penconazole)		(%50 Kresoxim-ethyl)
			25 mL/100 L		20 mL/100 L
	Fifth spraying	June 7	Prostar	June 24	Topas
			(%50 Kresoxim-ethyl)		(100 g/L Penconazole)
			20 mL/100 L		25 mL/100 L
	Sixth spraying	June 24	Topas	July 8	Prostar
			(100 g/L Penconazole)		(%50 Kresoxim-ethyl)
			25 mL/100 L		20 mL/100 L
	Seventh spraying	July 13	Prostar	July 26	Topas
			(%50 Kresoxim-ethyl)		(100 g/L Penconazole)
			20 mL/100 L		25 mL/100 L
	Eighth spraying	August 9	Rage	August 12	Rage
			(%70 Thiophanate-ethyl)		(%70 Thiophanate-ethyl)
			100 g/100 L		100 g/100 L

**Table 3:** Fungicides used in the DSS model spraying program in 2021–2022 and application numbers

Spray model	Number of spray	2021	2022	2021	2022
		Date of spraying	Name of the fungicide (ingredient and dosage)	Date of spraying	Name of the fungicide (ingredient and dosage)
DSS model	First spraying	April 14	Burgundy slurry	April 25	Burgundy slurry
			(20% metallic copper)		(20% metallic copper)
			500 g/100 L		500 g/100 L
	Second spraying	April 30	Copper	May 26	Topas
					(100 g/L Penconazole)
			500 g/100 L		25 mL/100 L
	Third spraying	May 24	Topas	June 10	Topas
			(100 g/L Penconazole)		(100 g/L Penconazole)
			25 mL/100 L		25 mL/100 L
	Fourth spraying	June 22	Topas	June 24	Prostar
			(100 g/L Penconazole)		(%50 Kresoxim-ethyl)
			25 mL/100 L		20 mL/100 L
	Fifth spraying	July 13	Prostar	—	—
			(%50 Kresoxim-ethyl)		
			20 mL/100 L		

### 2.2 Spray models

The trial plots were designed to consist of six vineyards in a row, in accordance with the model, and phenology studies, spraying, counting, and evaluations were conducted in the middle four vineyards. The study used a randomized block trial design. During spraying operations, plastic safety sheets were used for controlling chemical transfer between the experimental plots. Using a randomized block trial design with four blocks and four replications each, the study was carried out using four different applications: the UC Davis risk index model, the DSS model (Mildium^®^ model), the spraying according to phenology model (the model recommended by the Ministry of Agriculture and Forestry), and control.

**UC Davis powdery mildew risk assessment model**: A portion of the model forecasts ascospore release based on leaf wetness and temperature [9].

Ascospore infection forecasts:

- Predictions are based on average temperature during an extended leaf wetness event.
- The model utilizes the “Conidial Mills Table” at 2/3 value for hours of leaf wetness required at various temperatures.
- Generally, at least 12–15 h of continuous leaf wetness is required when average temperatures are between 10 and 15°C.

Once infection has occurred, the model switches to the risk assessment phase, which is based entirely on the effect of temperature on the reproductive rate of the pathogen. The risk assessment model is described in the table below.

University of California grapevine powdery mildew risk index for conidial increase.

Conidial Infections – Risk Assessment (Risk index ranges from 0 to 100).

- Requires 3 consecutive days with at least 6 h between 21 and 30°C to trigger the index.
- The index increases 20 points each day with at least 6 h between 21 and 30°C.
- The index decreases 10 points each day with less than 6 h between 21 and 30°C.
- The index decreases 10 points on any day with a minimum temperature above 35°C.
- An index of 60–100 indicates the pathogen is reproducing every 5 days.
- An index of 0–30 indicates the pathogen is functioning minimally and the reproductive rate is every 15 days or not at all.

**DSS model:** The DSS model for powdery mildew was constructed to restrict the cluster damage to a threshold of 5% severity at harvest time [21] without considering its level on leaves. To implement the DSS, three indices were recorded at different phenological stages as follows [22]:

- The occurrence of signs on leaves was assessed visually for every 20 vines in the field (eight leaves per plant) at the growth stage of single flower separated (code 12 of the modified Eichhorn and Lorenz scale [E–L scale]) [23].
- The occurrence of signs on clusters was evaluated by visually observing all clusters of approximately 40 vines per hectare at 24 days after flowering. This evaluation aimed to determine whether a new fungicide application was required subsequent to the two obligatory applications.
- The persistence effect of the fungicide active ingredient was used to determine the maximum time required between the two treatments.

The cultivation period was established and continued for 2 years. The same essential cultural practices for growing were applied to control plots and other applications. Details of the fungicides used in 2021–2022 are given in Tables 1–3. Additionally, the names of the fungicides used and their spraying intervals according to the risk index are given in Table 4.

**Table 4:** Names of fungicides used and spraying intervals according to the risk index

Risk index	Disease severity	Status of the pathogen	Recommended spraying program		
			Sulfur	DMI inhibitors	Strobilurins and quinolines
0–30	Low	Available	14–21-day intervals	21 days or the interval specified on the label	21 days or the interval specified on the label
40–50	Middle	Reproduces every 15 days	10–17-day intervals	21-day intervals	21-day intervals
>60	High	Reproduces every 5 days	7-day intervals	10–14-day intervals	14-day intervals

### 2.3 Calculating the incidence and severity of the disease

During the study period, census and evaluation studies were carried out to determine disease severity and incidence rates. The leaf and bunch evaluation and counting procedures followed the recommendations of Plant Diseases Standard Drug Trial Methods [24] for powdery mildew disease. For both grape varieties, assessments were conducted on the leaves and groups of four vines centered at approximately six vines in each parcel.

**Leaf evaluation:** A total of 100 leaves from four vines were collected using the Plant Diseases Standard [24] scale; 25 leaves were randomly selected from the surrounding shoots of each vine to be counted after the third leaf from the bottom. Furthermore, the percentage disease incidence rate was calculated by dividing the total number of investigated leaves by the number of infected leaves, regardless of the number of spots, to calculate the disease incidence rates on the leaves [25]. The evaluation scores for powdery mildew disease on leaves are shown in Table 5.

**Table 5:** Evaluation score of powdery mildew disease in leaves

Score	Description
0	There are no spots on the leaf
1	1–2 spots on a leaf
2	3–10 spots on a leaf
3	>10 spots on a leaf

**Evaluation in bunches:** Diseased and healthy berries were counted in a total of 20 clusters, including at least five bunches of the four vines in the middle of each parcel, and the % disease incidence rate was determined by proportioning the number of diseased berries in the parcel to the total number of examined berries. Evaluations were made after the last spray, when the duration of effect of the spray and the incubation period of the disease agent (5–6 days) had passed, and when the disease rate in the control was 20% or greater.

From the scale values acquired from leaf counts, percentage disease severity was determined using the Townsend-Heuberger [26] formula; the Abbott [27] formula was then used to calculate the percentage effect of spraying models from these values. Furthermore, the Abbott formula was used to calculate the percentage impact of spraying models based on the disease incidence rates in diseased leaves and bunches [24].

### 2.4 Analysis of pesticide residues

Pesticide residue analyses, comparisons between spraying models, and comparisons of adherence to the residual limitations of the Turkish Food Codex were performed on preharvest samples of the Michele Palieri and Muscat Hamburg varieties cultivated in the trial region. Following the final fungicide application in the spraying programs, grape samples were collected by paying attention to the preharvest waiting period. For sampling, grape bunches were randomly selected from each parcel, and distinct sampling procedures were used for each variety of grape. Two kilogram samples were then taken and submitted to the laboratory on the same day. Analyses of residues were conducted using the methodology outlined by the AOAC International [28].

The extraction of nonpolar pesticides was performed using the QuEChERS kit in accordance with the manufacturer’s instructions. Five hundred grams of grape fruit samples contaminated with pesticide standards were homogenized in a Retsch brand GM300 model blender. Fifteen grams of each of these homogenized samples was placed in a 50 mL polypropylene centrifuge tube, and 15 mL of acetonitrile + 1% acetic acid was added. Then, 6 g of anhydrous magnesium sulfate and 1.5 g of sodium acetate (QuEChERS extract pouch AOAC, Agilent 5982-7755) were added to the tubes and shaken vigorously for 1 min. Subsequently, the tubes were centrifuged at 4,000 rpm for 1 min, and the upper layer (8 mL) in the tubes was carefully withdrawn with a pipette, and 1,200 mg of anhydrous magnesium sulfate and 400 mg of PSA (QuEChERS Dispersive Kit, General Fruits and Vegetables, AOAC method, Agilent 5982-5058 5982-5650CH) were transferred to a 15 mL Falcon tube. After the tubes were vortexed for 30 s and centrifuged at 4,000 rpm for 1 min, the upper layer was filtered with a 0.45 µm diameter membrane filter (Bond Elut Hydrophobic 17 576 filter, Agilent). Pesticide concentrations were measured using an Agilent 6470 Triple Quad Liquid-Mass Spectrometer (Agilent Technologies, Santa Clara, CA, USA). An Agilent Poroshell SB-C18 column (3 mm × 100 mm × 2.7 µm) was used for chromatographic separation. The mobile phase consisted of 0.1% formic acid and 1 mM ammonium formate solution and an aqueous solution containing ultrasonically pure water (A) and methanol (B). The mobile phase flow rate was set to 0.52 mL/min, the gradient elution was 0–0.5 min at 70% A, 0.5–8 min at 70% A, 8–12.5 min at 5% A, and 12.5−15.0 min at 70% A. Detection by mass spectrometry (MS) was performed in multiple reaction monitoring (MRM) and electrospray ionization (ESI) mode. The gas flow was set to 10 psi, the gas capillary voltage was set to 3,600 V, the source temperature was set to 100°C, and the sample injection volume was set to 1 µL.

### 2.5 Statistical analyses

Each variant was examined by analysis of variance to determine significant differences among applications. The experiment was carried out in three replicates on randomized experimental design plots for 2 years. For each variant, LSMeans differences Student’s test (minimum significant difference method) was used to determine the level of resistance for all accessions. Duncan’s multiple range test was used to compare infections. Significant differences were defined as those for which *p* <0.05. Pearson’s correlation coefficient (*R*) was used to evaluate covariance relationships between variables. The JMP 7.0 program was used for all analyses [29].

## 3 Results

### 3.1 Effectiveness of spray models

During the 2021 and 2022 growing seasons, the leaves and berries of the Muscat Hamburg and Michele Palieri grape varieties were used to assess the efficacy of the spraying treatments. Disease assessments on leaves and bunches were conducted in both production seasons following the final spraying, the chemical effect period, and the disease agent’s incubation period. Particularly when disease evaluations were made, scores were made by taking into account the diseased areas on both leaves and bunches [24].

### 3.2 Effectiveness of foliar spraying models

**Figure 3:**
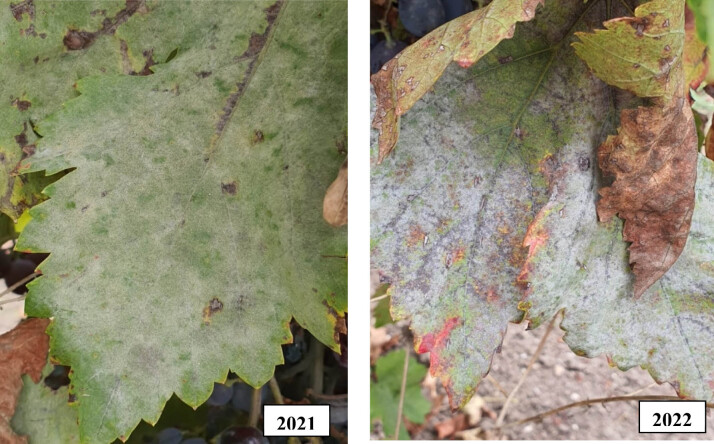
Photos of average disease severity in leaves in the 2021 and 2022 seasons.

The classical model, UC Davis risk index model, and DSS model spraying techniques were compared for their degree of leaf powdery mildew disease control in the experimental plots containing the Muscat Hamburg and Michele Palieri grape varieties in the study performed to control the disease on grapevines. When the control plots exhibited a 20% increase in disease severity over both production seasons, an assessment was conducted to determine the efficacy of the spraying treatments on the leaves. Figure 3 shows the symptoms of powdery mildew that were observed on grape leaves in the experimental plots. Disease symptoms on the leaves were more prevalent in the 2022 production season than in the 2021 season, possibly as a result of climate factors (Figures 2a, b and 3).

Leaf disease severity in the control plots of the Michele Palieri grape variety was 57% during the 2021 growing season. The disease severities were 5.60, 10.8, and 30.58%, respectively, as a result of the pesticides used in accordance with the UC Davis risk index model, classical model, and DSS model pesticide methods. Powdery mildew disease can be prevented more successfully with the classic model and the UC Davis risk index model, as demonstrated in Table 6. To prevent powdery mildew, spraying strategies based on the UC Davis risk index model, Classic model, and DSS model were found to be 90.18, 82.32, and 46.35% effective in preventing the disease, respectively.

**Table 6:** Effect (%) and disease severity (%) of spraying methods applied to the Michele Palieri and Muscat Hamburg grape varieties in the 2021 and 2022 production seasons for preventing powdery mildew disease on the leaves*

Variety	Year	UC Davis risk index model		Classic model		DSS model		Control
		Disease severity (%)	Effectiveness of spray model (%)	Disease severity (%)	Effectiveness of spray model ( %)	Disease severity (%)	Effectiveness of spray model ( %)	Disease severity (%)
Michele Palieri	2021	5.60c	90.18	10.08c	82.32	30.58b	46.35	57.00a
Michele Palieri	2022	18.63c	77.71	16.00c	80.86	50.93b	39.06	83.58a
Muscat Hamburg	2021	1.10c	95.36	0.00c	100.00	6.58b	72.24	23.70a
Muscat Hamburg	2022	3.64d	90.70	8.18c	79.11	15.02b	61.64	39.16a
*p* value		<0.01	<0.01	<0.01	<0.01	<0.01	<0.01	<0.01

*Different small letters within the same column indicate that the means are significantly different (*p* < 0.01).

According to assessments conducted on the Michele Palieri grape variety during the 2022 growing season, 83.58% of the leaves in the control plots had disease. In this case, the disease severity in the parcels treated with the UC Davis risk index model, classical model, and DSS model were 18.63, 16.00, and 50.93%, respectively, for the pesticides administered in accordance with various pesticide methods. The efficacy rates of the spraying strategies used to prevent powdery mildew based on the UC Davis risk index model, Classic model, and DSS model were 77.71, 80.86, and 39.06%, respectively. Despite the less performance compared to 2021, the UC Davis risk index model and the Classic model appear to be more successful in preventing powdery mildew in 2022 (Table 6).

During the 2021 growing season, field research on the Muscat Hamburg grape variety revealed that the disease severity on the leaves in the control plots was 23.70%. Disease severity on the leaves as a result of spray changes according to the DSS model, classical model, and UC Davis risk index model was 1.1, 0, and 6.58%, respectively. When the classic model was sprayed on the leaves in 2021, no signs of disease were observed. This may be due to the effectiveness of the spraying model, as well as the low disease pressure in 2021. The effectiveness rates of the UC Davis risk index model, classic model, and DSS models applied in 2021 for powdery mildew were determined to be 95.36, 100, and 72.24%, respectively (Table 6).

The leaf disease severity of the Muscat Hamburg grape variety increased to 39.16% in the control plots during the 2022 grape spraying season. In this study, the disease severities were 3.64, 8.18, and 15.02%, respectively, in the experimental plots when the UC Davis risk index model, classical model, and DSS model were employed. The data obtained in the control plots indicate that, similar to the Michele Palieri grape variety, the disease pressure is greater in 2022 than in 2021 (39.16% in 2022 and 23.70% in 2021). After analyzing the data from the 2 years, it was determined that the classic model and the UC Davis model outperformed DSS in preventing powdery mildew (Table 6).

### 3.3 Effectiveness of spraying modes in bunches

The effectiveness levels of the classical model, UC Davis risk index model, and DSS model spraying methods were determined as part of the study conducted in the 2021 and 2022 cultivation seasons to prevent powdery mildew disease in the clusters of Michele Palieri and Muscat Hamburg varieties. For the Michele Palieri grape variety, the disease rate was 24.07% during the 2021 production season, but it decreased to 21.8% during the 2022 season. It was found to be 7.30 and 12.82% in the Muscat Hamburg grape variety in the 2021 and 2022 production seasons, respectively (Table 7).

**Table 7:** Effect (%) and disease severity (%) of spraying methods applied to the Michele Palieri and Muscat Hamburg grape varieties in the 2021 and 2022 production seasons for preventing powdery mildew disease on bunches*

Variety	Year	UC Davis risk index model		Classic model		DSS model		Control
		Disease severity (%)	Effectiveness of the spray model (%)	Disease severity (%)	Effectiveness of the pray model (%)	Disease severity (%)	Effectiveness of the spray model (%)	Disease severity (%)
Michele Palieri	2021	2.32d	90.36	5.32c	77.90	11.40b	52.64	24.07a
Michele Palieri	2022	2.22d	89.82	5.77c	73.53	9.80b	55.05	21.80a
Muscat Hamburg	2021	4.38c	40.07	5.10bc	30.14	6.40ab	12.33	7.30a
Muscat Hamburg	2022	1.15c	91.03	1.35c	89.47	2.62b	79.56	12.82a
*p* value		<0.01	<0.01	<0.01	<0.01	<0.01	<0.01	<0.01

*Different small letters within the same column indicate that the means are significantly different (*p* < 0.01).

While the disease incidence rate in Michele Palieri cluster in control was 24.07% in the 2021 production season, the disease incidence rates in the spraying models used by the UC Davis risk index model, classical model, and DSS model were found to be 2.32, 5.32, and 11.4%, respectively. These results indicated that the UC Davis model outperformed the other models. As shown in Table 7, the efficacy rates of the powdery mildew disease spraying methods based on the UC Davis risk index model, Classic model, and DSS model were 90.36, 77.90, and 52.64%, respectively, in preventing disease in bunches.

The disease incidence rate in the control was slightly lower (21.8%) than that in the 2021 season, based on observations and analyses conducted on the clusters of the Michele Palieri grape variety in 2022. The rates of disease incidence in parcels sprayed with the DSS model, classical model, and UC Davis risk index model were found to be 2.22, 5.77, and 9.8%, respectively. The powdery mildew prevention effectiveness of spraying strategies based on the UC Davis risk index model, Classic model, and DSS model in bunches was found to be 89.82, 73.53, and 55.05%, respectively. These results indicated that the UC-Davis risk index model outperformed the other models in terms of effectiveness. The Classic model’s effectiveness was marginally lower than the 2021 effectiveness figures, even though the UC Davis risk index model once more attained a high degree of effectiveness. The DSS model’s target of maintaining the disease prevalence rate in fruits below 5% was not achieved, as in 2021 (Table 7).

According to the results of the observations and analysis performed on the Muscat Hamburg grape variety bunches in the 2021 production season, the disease prevalence rate in the control was determined to be very low at 7.30%. Although there is no need to count according to the Plant Diseases Standard [24], disease prevalence rates for bunches have been determined according to the UC Davis risk index model, classical model, and DSS model. According to these census, the disease prevalence rates were 4.37, 5.10, and 6.40%, respectively. According to the census and evaluations of clusters for Muscat Hamburg in 2021, the DSS model again failed to meet the maximum 5% disease incidence rate criterion. The effectiveness rates of the UC Davis risk index model, classical model, and DSS model in preventing disease in bunches were 40.07, 30.14, and 12.33%, respectively. According to these results, although the UC Davis risk index model stands out compared to other models in terms of effectiveness, it still showed a very low effectiveness percentage. Similarly, the classical model also showed a very low percentage of effectiveness (Table 7).

According to the evaluation of bunches of the Muscat Hamburg grape variety in the 2022 production season, the disease prevalence rate in the control plots was 12.82%, which was similar to that in the 2021 season. Once more, all rates have been established even though the Plant Diseases Standard [24] does not specify the disease prevalence rates. Clusters in plots where spraying methods were based on the DSS model, classical model, and UC Davis risk index model yielded disease prevalence rates of 2.62%, 1.35%, and 1.15%, respectively. These results suggest that the UC-Davis risk index model performed better than the other models. The rates of success of the spraying strategies based on the UC Davis risk index model, classical model, and DSS model in preventing bunch disease were very high at 91.03, 89.47, and 79.56%, respectively (Table 7).

### 3.4 Harvest period residue analysis

Table 6 presents the residue amounts in grapes during harvest in the experimental plots for both production seasons based on the active ingredients within the parameters of the study. The active component 70% thiophanate-methyl (Rage) was found to be above the MRL value (0.1 mg/kg in the Turkish Food Codex) for the 2021 production season using the classic model and UC Davis risk index model for both grape varieties. It was found that the value was above the limit even though all models observed the specified 14-day waiting period for 70% thiophanate-methyl between the last spraying and the harvest time. For both grape varieties, the residual limits for the 70% thiophanate-methyl (Rage) active component in all spraying methods did not exceed those of the 2022 production season and remained below the limit values.

The thiophanate-methyl residue level for the grape variety Michele Palieri was found to be 0.497 mg/kg in samples taken from parcels sprayed with the classic model in 2021. This value is approximately five times greater than the TGK MRL standard of 0.1 mg/kg. The residue level in the samples was found to be 0.613 mg/kg when the same active ingredient was sprayed using the UC Davis risk index model, which is more than six times greater than the TGK MRL value of 0.1 mg/kg. The amount of thiophanate-methyl residue in the samples from 2021 sprayed with the Muscat Hamburg grape variety using the classical model was 0.989 mg/kg, which was approximately ten times greater than the TGK MRL value of 0.1 mg/kg.

Table 8 shows the maximum residue found in the studies of 50 g/L kresoxim-methyl (Prostar) and 100 g/L penconazole (Topas) fungicides sprayed on the Muscat Hamburg and Michele Palieri grape varieties in both production seasons using the classic model, UC Davis risk index model, and DSS model. There were no samples determined to be over the limits.

**Table 8:** Residue analysis results of berries taken from different spray treatments

Year	Variety	Active ingredient	Control (mg/kg)	Classic model (mg/kg)	UC Davis model (mg/kg)	DSS model (mg/kg)	TGK** MRL (mg/kg)
2021	Michele Palieri	Thiophanate-methyl	0.012	0.497	0.613	0.006	0.1
		Kresoxim-methyl	ND*	ND	0.006	0.002	1.5
		Penconazole	ND	ND	ND	ND	0.05
	Muscat Hamburg	Thiophanate-methyl	0.023	0.989	1.292	0.015	0.1
		Kresoxim-methyl	ND	0.002	0.001	ND	1.5
		Penconazole	ND	ND	ND	ND	0.05
2022	Michele Palieri	Thiophanate-methyl	ND	ND	ND	ND	0.1
		Kresoxim-methyl	ND	ND	ND	ND	1.5
		Penconazole	ND	ND	ND	ND	0.05
	Muscat Hamburg	Thiophanate-methyl	ND	0.012	ND	ND	0.1
		Kresoxim-methyl	ND	ND	ND	ND	1.5
		Penconazole	ND	ND	ND	ND	0.05

*ND: not detected. **TGK MRL: Maximum residue limits according to the Turkish Food Codex.

## 4 Discussion

Three alternative control strategies were applied to Michele Palieri and Muscat Hamburg grape varieties for 2 years, and the results were comprehensively compared with those of researchers who had conducted similar studies.

In addition, the amount of pesticide residue left in the final product by the spraying models used for control purposes was determined, and all results were compared with each other and checked if they are within the legal residue limits. The evaluation of the 2021 and 2022 production seasons indicated a notable increase in disease severity in the control plots for both the Muscat Hamburg and Michele Palieri grape varieties. Researchers reported that climate conditions that change from year to year, especially unstable rainfall, are one of the main factors in the changes in the severity of fungal diseases. Increased leaf wetness periods have been linked to an increase in disease pressure, particularly when there is an increase in rainfall and insufficient sun exposure [10,30,31].

The results of spraying studies using the classical model in this study were comparatively better than those of the study on the Sultani grape variety in Manisa Province by Savaş et al. [32], which suggested several spraying techniques based on phenological development stages. In their study, the researchers found that the classical model, with six sprayings, was 53.88% successful on the leaves. At the same time, the other two suggested phenology-based models, with seven and nine sprayings, were 69.17 and 73.79% effective, respectively. In our study using the classical model, 77.90% effectiveness was reached in our spraying using the classical model in the 2021 production season (24.07%), when the disease prevalence rate in Michele Palieri bunches was the highest. With a few modifications and sensible chemical (active ingredient) selections, the outcomes we found using the classical model might offer greater efficacy against grapevine powdery mildew.

Bakırcı et al. [33] reported that fungicides containing thiophanate-methyl as the active component may result in residual issues in vineyards based on their thorough residue analysis of fruits and vegetables in the Aegean region. In our study, the active ingredient 70% thiophanate-methyl in both grape varieties in the 2021 production season exceeded the MRL of the Turkish Food Codex, which is consistent with previous studies regarding the active ingredient in question.

Similar to the results obtained in our study, in residue analysis studies with table grapes in Türkiye, Gölge and Kabak [34] reported that the active ingredients kresoxim-methyl and penconazole did not cause residue problems. A study conducted by Poulsen et al. [35] in Denmark reported that residue limits were not exceeded in samples treated with penconazole. In another residue study conducted on wine grapes in Slovenia by Baša Česnik et al. [36], the authors reported that the active ingredient kresoxim-methyl did not exceed the MRL. These results do not exceed the MRLs of the active ingredients kresoxim-methyl and penconazole and are similar to the results of our study. In parallel with the results obtained in our study, researchers [20] also reported that reducing the spraying interval increases the protection but increases the cost and risk of residue. In addition, it is observed that there are differences between years in terms of resistance to powdery mildew, as well as between grape varieties. Researchers have also reported this situation in studies conducted with different grape varieties [37].

Lu et al. [38] used a model-guided fungicide spray method to predict the ideal spraying time for powdery mildew disease. Using an operational forecast model with supervised and algorithm model learning and integrating Global Forecast System (GFS) Ensemble Reforecasts (GEFSR) is highly helpful in providing effective protection, according to their study’s results. Future research can find that using various artificial intelligence technologies will greatly increase the efficacy of pesticide programs.

## 5 Conclusions

The agriculture sector, like all other sectors, experiences a number of issues that arise as a result of climate change. Plant disease pressure is on the rise, particularly due to changing climatic conditions, and spraying programs are not effective in providing the desired outcomes. The majority of pesticides are used for treating diseases, particularly those of fungal origin. Powdery mildew disease is among the most common fungal diseases in vineyards, and pesticides are often applied to protect grapevines and bunches in vineyards. Our research revealed that, in terms of controlling powdery mildew disease, different spraying methods may vary based on variety and year. Therefore, developing various spraying models for different product groups and varieties and determining which one works best can not only lower the amount of chemicals used but also greatly reduce the risk of residue. One of the active ingredients in our research, thiophanate-methyl, raised issues with residue limitations. Therefore, more studies on fungicides containing thiophanate-methyl as the active ingredient are needed. Its usage in agriculture may be limited, as in some countries, or its timing may be modified. Additionally, alternating the application of fungicides with different active ingredients can help prevent residue issues, lower the risk of pathogen resistance to fungicides, and achieve a sufficient level of protection against powdery mildew compared to applying a single fungicide at regular intervals throughout the season, as demonstrated in this study. According to the results obtained from this study, more reliable data can be obtained by testing programs for more grape varieties in different regions. It also becomes clear that policymakers need to introduce new laws that are more deterrent to pesticide residues. The public’s awareness of environmental and human health is growing every day. Therefore, for sustainability, it is crucial to stop spray programs that pose the danger of residue and develop spray programs that offer better protection with fewer pesticide applications. Future research should focus on using organic pecticides, and efforts should be made to reduce the possibility of chemical removal. In addition, studies where artificial intelligence is used more will contribute significantly to the sustainable viticulture model.
